# Death and the Devil

**Published:** 1870-07

**Authors:** 


### DEATH AND THE DEVIL.

Some workmen at Mooresville, Ind., have made a discovery, which goes very far toward settling the question whether there is a personal devil or not, though we are at loss to tell on which side the question is settled. In making an excavation, they dug up a skeleton well preserved, corresponding to the human skeleton in all respects, except that the forehead was villainously low, with two horns curving backward; the arms were of unusual length and the spinal bone terminated in a tail, of which about a foot remains. Monkeyism would account for the tail, but fails lamentably on the horns. Now arises the interesing question, whether, if there be anything Satanic about this skeleton, it proves the original demon to be dead or alive.—*Phila. Press.*

SHE DID.—"John," asked a medical friend of his office boy, "did Mrs. Jones get the Medcine I put up this morning for her?" "I guess so," replied John, "for I saw crape on the door-knob this morning!"

